# In Silico Reconstitution of *Listeria* Propulsion Exhibits Nano-Saltation

**DOI:** 10.1371/journal.pbio.0020412

**Published:** 2004-11-30

**Authors:** Jonathan B Alberts, Garrett M Odell

**Affiliations:** **1**Center for Cell Dynamics, University of WashingtonFriday Harbor, WashingtonUnited States of America

## Abstract

To understand how the actin-polymerization-mediated movements in cells emerge from myriad individual protein–protein interactions, we developed a computational model of Listeria monocytogenes propulsion that explicitly simulates a large number of monomer-scale biochemical and mechanical interactions. The literature on actin networks and L. monocytogenes motility provides the foundation for a realistic mathematical/computer simulation, because most of the key rate constants governing actin network dynamics have been measured. We use a cluster of 80 Linux processors and our own suite of simulation and analysis software to characterize salient features of bacterial motion. Our “in silico reconstitution” produces qualitatively realistic bacterial motion with regard to speed and persistence of motion and actin tail morphology. The model also produces smaller scale emergent behavior; we demonstrate how the observed nano-saltatory motion of *L. monocytogenes,* in which runs punctuate pauses, can emerge from a cooperative binding and breaking of attachments between actin filaments and the bacterium. We describe our modeling methodology in detail, as it is likely to be useful for understanding any subcellular system in which the dynamics of many simple interactions lead to complex emergent behavior, e.g., lamellipodia and filopodia extension, cellular organization, and cytokinesis.

## Abstract 2

A detailed computer simulation explicitly simulates monomer- scale biochemical and mechanical interactions to characterize bacterial motion

## Introduction

Cellular processes generally involve interactions among 10^1^ to 10^5^ gene products. These interactions can be both biochemical, as in the activation of one protein by another, and mechanical, as in the application of force between bodies. Even when each individual interaction is simple and understood in detail, neither intuition nor qualitative description can forecast the emergent behavior of the whole system. We describe a methodology to characterize such emergent behavior using a detailed computer simulation of both biochemical kinetics and mechanical dynamics. In this paper, we apply the technique to the motility of the bacteria *Listeria monocytogenes,* a well-studied system in which actin network growth produces a force that moves the bacterium inside of cells. We discuss the model design, compare behaviors of the computational and biological systems, use the model to explain observed features of the bacterial motion, and identify observable experimental correlates of our hypotheses through which our interpretations may be confirmed or rejected.

L. monocytogenes is a pathogenic rod-shaped bacterium that invades cells, reproduces, and spreads to neighboring cells, never exposing itself to the extracellular environment, thus avoiding a humoral immune response (Tilney and Portnoy 1989). By expressing the protein ActA (Domann et al. 1992; Kocks et al. 1992, 1995), L. monocytogenes bypasses the host cell's normal controls on actin network growth to produce a dense “comet tail” of actin. This actin tail generates a ram force, by rectifying thermal motion, to both propel the bacterium within a cell and push the bacterium into neighboring cells through distension of the cell plasma membranes.

Among experimental advances thus far made to understand this motile system are identification of the purified proteins required to reconstitute motion in vitro (Loisel et al. 1999), an ability to mimic this motion using polystyrene beads coated with the bacterial ActA protein (Cameron et al. 1999, 2001, 2004), and experiments that have revealed a discrete step-like motion on the nanometer scale (Kuo and McGrath 2000; McGrath et al. 2003). A series of complementary theoretical models have been proposed to account for some observed features of bacterial and bead motion (Peskin et al. 1993; Mogilner and Oster 1996, 2003; Gerbal et al. 2000a, 2000b; van Oudenaarden and Theriot 2000). These studies, taken together, show that *L. monocytogenes'* actin structures, first described by Tilney and Portnoy (1989), are created from the same protein components and perform a function similar to the actin machinery in the lamellipodia of motile cells. The dendritic nucleation model for actin network growth (Mullins et al. 1998; Pollard et al. 2000, 2001; Pollard 2003) offers a qualitative description of this biochemical network. In the absence of the bacterium, specific signals activate WASP/Scar proteins, and these in turn activate the Arp2/3 protein complex to provide new filamentous actin (F-actin) nucleation sites at or near the barbed (plus) end of existing filaments (Higgs and Pollard 2001). These new filaments form at a characteristic 70° angle to the parent filament, creating dense, highly branched networks (Mullins et al. 1998). Filament barbed ends are rapidly capped with high affinity by capping protein, making the creation/maintenance of free barbed ends critical for continued network growth. Hydrolysis of the ATP that was bound to each actin monomer favors filament disassembly, returning actin monomers to the pool of polymerization-ready G-actin. Cofilin aids in this disassembly by fragmenting F-actin, binding with much higher affinity to ADP actin than to ATP or ADP-Pi actin. The motion of L. monocytogenes exploits all of these actin network features, except that the bacteria's ActA replaces the host cell's WASP/Scar proteins and all the associated upstream signaling mechanisms that normally activate WASP/Scar to control actin polymerization (Welch et al. 1998; Zalevsky et al. 2001).

Our model differs in several ways from previous attempts to generate mathematical or physical models for L. monocytogenes motility (though see Carlsson 2001, 2003). We simulate explicitly a large number of detailed interactions of both a biochemical and mechanical nature, representing all protein–protein binding interactions with on-rate and off-rate kinetic equations. The simulation of actin filament polymerization, for example, depends on the local concentration of actin monomers and the association and disassociation rate constants (which have been experimentally determined), modulated by the steric accessibility of free barbed ends. Together these factors determine the binding/dissociation probabilities for each filament at each simulation time-step. Bulk properities of our actin “gel” arise from the contributions of the many individual parts of the actin network. Our model can thus accomodate arbitrary geometries, explicit stochastic input, and specific small-scale events. Mechanical interactions, which resolve collisions and accommodate the stretching of protein–protein linkages, follow Newton's laws.

We can represent any particular interaction in as coarse or detailed a fashion as desired, subject to the availability of computer resources, and each of these can be based either on experimental information or on simple postulates. We can determine the emergent behavior of the system, which is the dynamical outcome of all the particular interactions, only by running the computer program for many hours or days. In such a model it is neither possible nor desirable to include all details. If our model fails to characterize experimentally observed behavior, then something is missing. If our model does capture an emergent behavior, however, then we can study how quantitative changes in the underlying details (e.g., protein concentrations or specific rate constants) affect this larger scale behavior. The exploration of putative mechanisms is also straightforward, as it is easy to add, remove, or modify each individual interaction.

With our approach, we formalize experimentally based models of specific protein–protein interactions and biochemical kinetics in a direct and flexible way, but there are drawbacks. The theoretical approaches used to analyze the Brownian ratchet model and its refinements (Peskin et al. 1993; Mogilner and Oster 1996, 2003) facilitate the derivation of equations that describe important system characteristics, such as force–velocity curves. No such equations are available in our stochastic, individual molecule-based model; instead, we must distill parametric relationships from ensembles of many repeated simulations. Completing these parametric studies in reasonable human time requires considerable computer resources.

The biochemical and mechanical interactions near the bacterial surface are stochastic processes involving hundreds of filaments. We model dynamic processes on a per filament basis, rather than through bulk network properties and average filament growth. The growth of any particular filament depends upon that filament's precise location, orientation, and biochemical state, all of which change through time. There is no better way to simulate such a system than with a model that tracks each of these variables for each individual filament. In the future, this type of detail will be essential to capture (and thus explain) many observed biological phenomena.

The trajectories generated by this model of L. monocytogenes motility display repeated runs and pauses that closely resemble the actual nanoscale measurements of bacterial motion (Kuo and McGrath 2000; McGrath et al. 2003). Further analysis of the simulation state at the beginning and ends of simulated pauses suggest a new interpretation of the experimental results. We show that there is no characteristic step-size or pause duration in these simulated trajectories and that pauses can be caused by both correlated Brownian motion and by synchronously-strained sets of ActA–actin filament mechanical links.

### The Model

**Figure 1:**
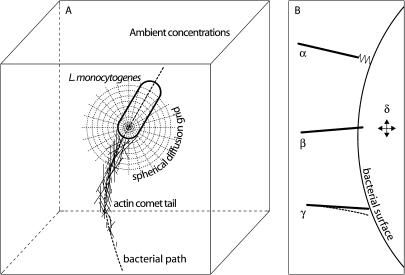
Model Schematic (A) shows a simple cartoon of the bacterium and some actin filaments *(explicit players)* against a backdrop of a moving diffusion–reaction grid attached to the bacterium. We use this spherical coordinate grid, whose origin moves with the bacterium, to keep track of the diffusion and biochemical interactions of the scalar protein concentrations fields that characterize implicit players. Protein size greatly impacts diffusion of proteins in a cellular environment (Luby-Phelps 1994, 2000), so we modify the nominal diffusion coefficients of implicit players accordingly. The dotted line is the path trajectory in 3D space of the bacterium. (B) illustrates the origin of forces that act on a bacterium in our simulation. Actin filament α is bound to an ActA protein on the bacterial surface, generating a link force that acts to hold the two objects together. Filament β is shown colliding with the bacterium, generating a collision force that acts to push the two objects apart. The barbed end of filament γ is nominally too close to bacterial surface to allow addition of an actin monomer, but Brownian motion might bend the filament into the dotted configuration, thus allowing polymerization and creating a collision. We model our filaments as rigid rods, but simulate this filament flexibility with a polymerization probability function that approaches zero with the filament–bacterium gap; i.e., it is possible for a filament to add a monomer even if the nominal filament–bacterium gap is less than 2.7 nm (the filament length increase per actin monomer). The bacterium, actin filaments, and structures of actin filaments are all subject to Brownian simulation forces and torques, represented by δ.

We model the molecular mechanics of the growth/disassembly of an actin network as it interacts with a moving rod-shaped bacterium to whose surface many ActA proteins are bound at specific locations. We distinguish molecular mechanics (Howard 2001) from molecular dynamics: we are not concerned with van der Waals forces and hydrogen bonds or with conformational changes during protein–protein interactions. Our model is different from a purely continuum model, in which state variables (those dependent variables which, together, fully describe the state of a system) would characterize only the bulk properties of an actin dense tail, using average compliance and polymerization values. We instead separate the cellular world into two basic classes of entities, those that are relatively large and present in small numbers (e.g., actin filaments, a bacterium) and those that are very numerous and small (e.g., actin monomers and other diffusible proteins). We simulate the former entities, which we call “explicit players,” as individuals; our state variables keep track of the position, orientation, and biochemical state of each individual and its change with time according to appropriate physical laws (e.g., Newtonian force balance laws). Those entities that are more numerous we will call “implicit players”: we represent them with continuum field state variables, i.e., molar concentrations that vary with time and place. We use standard partial differential reaction–diffusion conservation equations to express how these continuum fields change with time as the implicit players interact with each other and with the explicit (individual) players (Figure 1A). Dataset S1 is a simplified psuedo-code of our simulation software.

Various different forces impinge on the simulated bacterium. Forces move two objects apart if they happen to collide at the end of a time-step. Likewise, elastic bonds linking two objects (e.g., an actin filament–ActA bond) exert equal and opposite forces that hold those objects together; these links break under sufficient strain. Forces of random orientation act on every explicit player to simulate Brownian motion (i.e., the sum of all the many collisions with small molecules that, in biological reality, contribute to the Brownian motion is represented in our model by a single vector force and a single vector torque). This system never approaches an equilibrium; Brownian motion and biochemical events ceaselessly create collisions and perturb protein–protein links. Thus, we must compute new forces, exchanged between new neighbors, in each time-step. Figure 1B illustrates the set of mechanisms that combine to generate the net force on the bacterium in our simulation.

At the heart of this simulation is the dendritic nucleation model of actin dynamics (Mullins et al. 1998; Pollard 2003). Asynchronously, each individual actin filament can grow or shrink at either end by actin monomer polymerization/depolymerization; hydrolyze the ATP bound to one or more of its actin monomers to ADP-Pi; dissociate the Pi from one or more such monomers; be severed by ADF/cofilin; bind Arp2/3 to an ATP-actin subunit in the filament; be capped or uncapped at either end; and nucleate new filaments through Arp2/3 initiated side-branches. Repeated nucleation of new branches from existing filaments leads to a dense meshwork of actin in the comet-tail. Besides Arp2/3 mediated branching, all other cross-linking and adhesions involving actin filaments are implicit in the age and length dependent anchoring of f-actin in our simulation space. All actin filaments accumulate adhesions that gradually increase drag coefficients and eventually lock each filament into a fixed position. Figure 2, a video frame from a typical simulation, introduces some of the explicit and implicit players.

**Figure 2:**
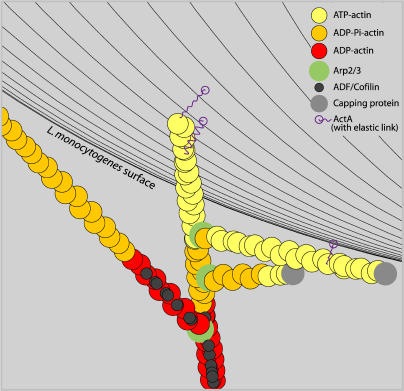
A Simulation Video Frame Showing Actin Filament Branching near the Bacterial Surface Arp2/3 seeds branches off of existing filaments at a characteristic 70° angle. Different ATP/ADP forms of actin have differing affinities for proteins such as ADF/Cofilin. Filament barbed tips can be capped unless they are protected through interaction with an ActA molecule; as indicated, surface-bound ActA molecules elastically link the bacterium to an actin filament near the barbed end.

The simulation time-step has a subtle effect on the simulation of Brownian motion for constrained objects (that is, objects linked to other objects). Applying the same forces and torques that are appropriate for free objects exaggerates the simulated Brownian motion of constrained objects since the motion restriction that results from those constraints can only be resolved over several time-steps, and those time-steps are large relative to the intrinsic time-scale of the constraints. Experimental measurements (Kuo and McGrath 2000) show very little Brownian motion (relative to similarly sized nearby vesicles) of L. monocytogenes associating with their actin tails; to match the biological reality, we need to modify our simulation of Brownian motion, since we cannot yet use much smaller time-steps. We compensate for this technical problem by carrying out simulations both for the two extreme cases (with Brownian motion appropriate for a free bacterium and with no Brownian motion of the bacterium at all) and for an intermediate degree of Brownian motion. Advances in computer processing speeds will, most likely, make such attenuation unnecessary in the near future. We will henceforth use the term “Brownian simulation force” to refer to the forces and torques that we apply to the bacterium to simulate its Brownian motion.

For our model, we use typical physiological concentrations for each of the proteins involved; these are listed in Table 1. Table 2 summarizes the reaction rate constants we used. Some crucial parameters and protein functions are as yet incompletely known. These include the exact pathways and rate constants associated with the stimulation of local actin filament polymerization by the bacterial surface-bound ActA protein. This protein has binding sites for a host of proteins, including G-actin, F-actin, Arp2/3, and Ena/VASP. In addition, while the affinity between free ActA and Arp2/3 has been measured (Zalevsky et al. 2001), that value (K_D_ = 0.6 μM) does not sufficiently characterize that interaction since the interaction rates may be limited by the flux of Arp2/3 or G-actin onto the surface. We have calculated the flux of Arp2/3 and G-actin onto the bacterium's surface to determine the expected equilibrium number of ActA–Arp2/3 and ActA–actin complexes there, as explained in Dataset S2. We tune these approximate rate constants to create actin networks with biologically representative side-branch separation and filament numbers. Because the rate constants that we have obtained in this way will depend on the concentrations of ActA, Arp2/3, and actin monomers in the model, the rates given for ActA–Arp2/3 and ActA–actin interactions in Table 2 apply to the concentrations given in Table 1.

**Table 1:**
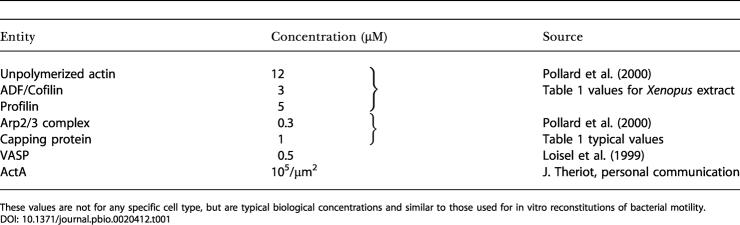
Values and References for the Concentrations of Proteins We Used

These values are not for any specific cell type, but are typical biological concentrations and similar to those used for in vitro reconstitutions of bacterial motility

**Table 2:**
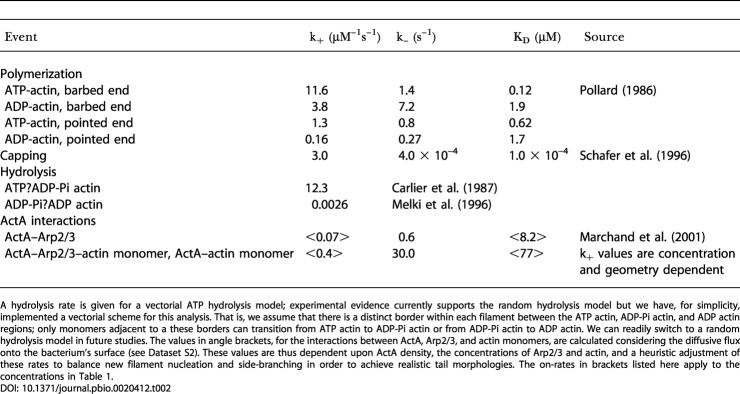
The Biochemical Rate Constants Incorporated in Our Model

A hydrolysis rate is given for a vectorial ATP hydrolysis model; experimental evidence currently supports the random hydrolysis model but we have, for simplicity, implemented a vectorial scheme for this analysis. That is, we assume that there is a distinct border within each filament between the ATP actin, ADP-Pi actin, and ADP actin regions; only monomers adjacent to a these borders can transition from ATP actin to ADP-Pi actin or from ADP-Pi actin to ADP actin. We can readily switch to a random hydrolysis model in future studies. The values in angle brackets, for the interactions between ActA, Arp2/3, and actin monomers, are calculated considering the diffusive flux onto the bacterium's surface (see Dataset S2). These values are thus dependent upon ActA density, the concentrations of Arp2/3 and actin, and a heuristic adjustment of these rates to balance new filament nucleation and side-branching in order to achieve realistic tail morphologies. The on-rates in brackets listed here apply to the concentrations in Table 1

The ActA protein is distributed asymmetrically on the bacterial surface, with more ActA near the rearward tail-forming end. The unipolar shape of our distribution is based on measurements of the fluorescence signal from RFP-labeled ActA along the bacterial length for newly divided bacteria (S. Rafelski and J. A. Theriot, unpublished data). New filaments are produced by two pathways. By activating Arp2/3, ActA is thought to catalyze the creation of new actin filaments and side-branches. We simulate the co-binding of ActA, Arp2/3, and an existing actin filament, allowing binding in any order. This complex leads to a new side-branch on the existing filament. Binding of ActA to the actin filament can occur only at specific ATP or ADP-Pi actin sites and is binding site limited, meaning that each bound ActA occludes a linear region of five monomers on the filament from further binding. Creation of a new filament de novo in our model involves the co-binding by ActA of G-actin and Arp2/3, in any order (Boujemaa-Paterski 2001).

In conjunction with other proteins (e.g., Ena/VASP), ActA may also regulate actin dynamics in other important ways (Goldberg 2001). In this version of our model, we do not explicitly simulate Ena/VASP molecules, which can regulate actin networks by binding profilin, competing with capping protein, and regulating Arp2/3 spacing (Krause et al. 2003). Instead we assume that ActA itself can uncap any actin filament barbed end to which it binds closely (within one ActA length) and ignore the other possible Ena/VASP functions. We find that this uncapping function is necessary to obtain persistent motion with a low nucleation rate of new filaments. For the values in Tables 1 and 2, our simulated bacterium do move without this uncapping, but more slowly. Loisel et al. (1999) have reported a similar dependence in their experiments, finding that Ena/VASP is not required for bacterial motion, but that it improves the efficiency of the motion observed.

## Results

**Figure 3:**
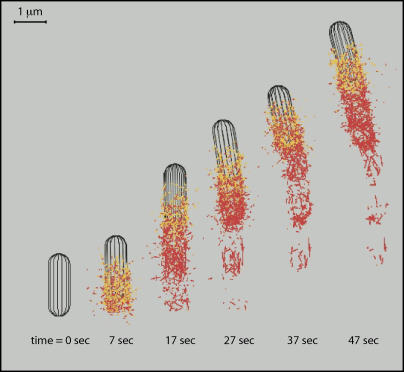
A Simulation Time Series Several video frames from one simulation show, on a gross scale, the de novo ActA filament nucleation and Arp2/3 branching from existing filaments that form a comet-tail, the hydrolysis of actin filaments in the comet-tail, and the subsequent pointed end depolymerization of ADP-actin (filament severing by ADF/Cofilin is not active in this simulation).

The gross behavior of our simulated bacterium is life-like; model bacteria move in a qualitatively similar way to wild-type L. monocytogenes. Average speeds of motion varied from ten to hundreds of nanometers per second, as do real observed bacterial speeds in different experiments (40 nm/s in purified proteins in Loisel et al. 1999; 140 nm/s in cytoplasmic extracts in Cameron et al. 2004; 1.4 μm/s in vivo in Dabiri et al. 1990). Videos at any scale may be rendered from our simulations (Videos S1 and S2; other full-length simulations at www.celldynamics.org). Figure 3 merges several frames from one of those movies, showing the large-scale formation, hydrolysis, and depolymerization of the actin comet-tail.

The microrheology experiments of Kuo and McGrath (2000) and McGrath et al. (2003) present an opportunity to illustrate the utility of our stochastic model founded on small-scale details. They reported that L. monocytogenes motility is multiphasic; motion of the bacterium that appears smooth on the micrometer length-scale actually consists of pauses that last many milliseconds, discrete nanometer-scale steps, as well as uninterrupted runs. No current model of this bacterial motility has fully explained these discrete steps, though numerical simulations with the tethered ratchet (Mogilner and Oster 2003) can exhibit saltatory motion. Possible explanations involving strained links between ActA and actin filaments and nucleotide dependent filament templates are discussed in McGrath et al. (2003).

**Figure 4:**
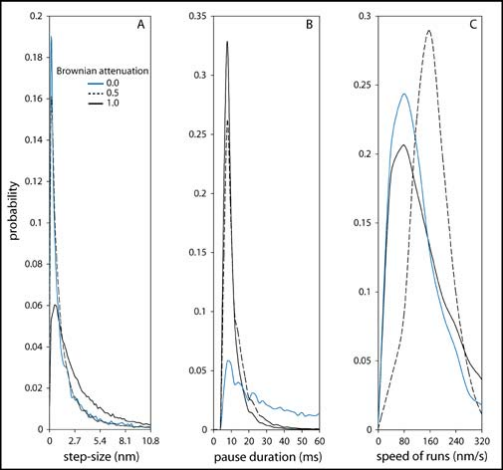
Step-Sizes, Pause Durations, and Speeds of Motion with Different Brownian Simulation Force Attenuation The legend shows the multiplier by which the Brownian simulation force on the bacterium is attenuated, such that a multiplier of 1 corresponds to a Brownian simulation force appropriate to an unconstrained bacterium and a multiplier of 0 signifies no simulated Brownian motion of the bacterium (see discussion of the relationship between applied Brownian simulation force and numerical time-step in the model description). We line-fit and filter trajectories by slope (speed) to identify pauses in the bacterial motion. Any line with slope less than the pause threshold might indicate a pause. The distance between adjacent pause locations is the step-size, assembled into a histogram in (A). No characteristic step-size is evident; smaller steps are simply more probable than larger ones. We exclude steps shorter than 0.2 nm. (B) shows a pause duration histogram; here we exclude pauses <8 ms in duration. (C) shows run speed histograms for runs >50 ms in duration. For a Brownian multiplier of 1, we used 30 simulations with 56,239 pause events for (A) and (B) and 6,098 runs for (C) (1,929 s total simulation time, 644 s total paused time, 116 nm/s average speed, pause threshold 40 nm/s). For a multiplier of 0.5, we used 13 simulations with 49,207 pause events and 2,287 runs (1,534 s total simulation time, 700 s total paused time, 93 nm/s average speed, pause threshold 30 nm/s). For a multiplier of 0, we used 18 simulations with 9,748 pause events and 1,179 runs (940 s total simulation time, 612 s total paused time, 56 nm/s average speed, pause threshold 20 nm/s).

Figure 4 shows the distribution of step sizes and pause durations that our model produces (using the values of Tables 1 and 2 and one set of mechanistic hypotheses) with three different assumptions for the Brownian motion of the bacterium (see under The Model). In all cases, we find that there is no characteristic step-size, but rather a continuum of steps with the smaller steps being more probable than larger ones. The qualitative shapes of these histograms are insensitive to changes in all parameters we might reasonably vary, barring values that disrupt persistent bacterial motion. The parameters we have varied include link characteristics (e.g., link length, link force, and maximum link strain), Arp2/3 branching rate, and temperature. In fact, even doubling the size of each actin monomer (easily done in a simulation) does not change these histograms significantly (data not shown).

These results suggest that our simulated bacterium does not move with steps of any prefered size, and specifically not with a step-size related to the actin monomer dimensions. The pause in forward progress might equally be considered the defining event in the bacterium's motion; a “step” in this case is just a run made along the path between adjacent pauses. But what physical process stalls the actin polymerization ram to initiate pauses, and what physical process breaks the bacterium out of each paused state into a run? To answer these questions, we need to examine how key descriptive system features vary before, during, and after pauses in our simulations. We do this by looking both at individual pauses and at the average of these system outcomes for many thousands of pauses (see Materials and Methods).

**Figure 5:**
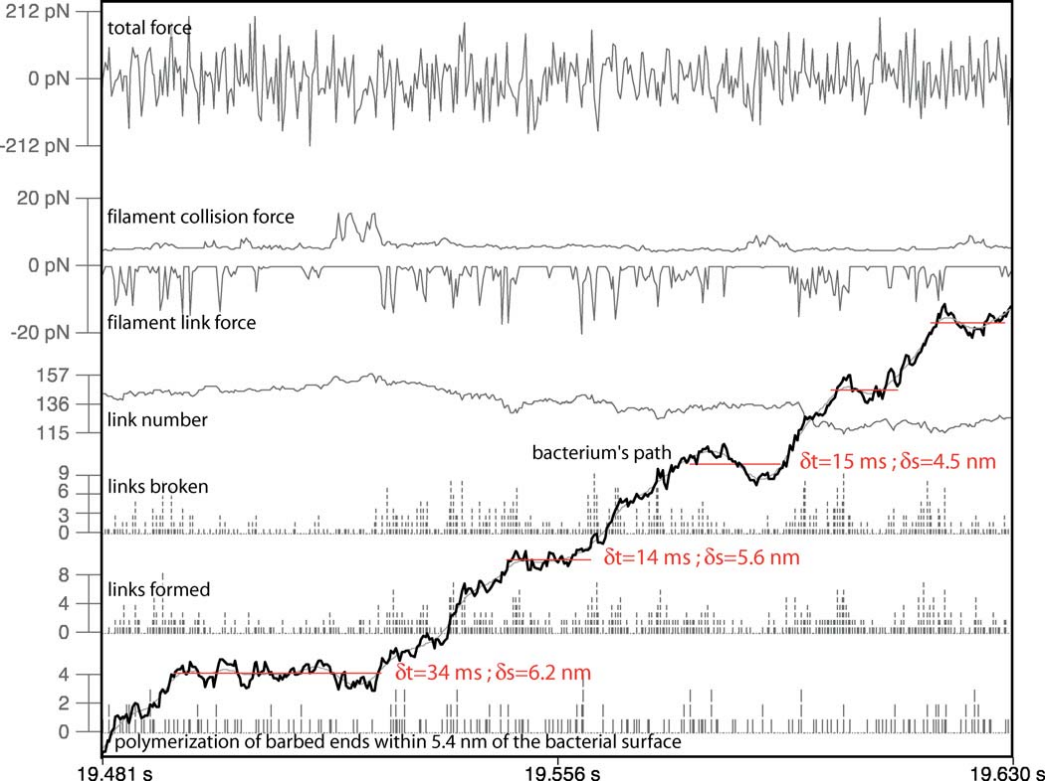
System Outcome Profiles for Several Adjacent Pauses Pauses are shown by red horizontal lines in a single simulation run in which the Brownian force multiplier was 1 (unconstrained bacterium). Listed for each pause are the pause duration (δt) and distance to the following pause (δs) as reported by our line-fitted analysis. The vertical line segments in the lower half of the figure show discrete events. From the bottom, these are the number of polymerization events on filaments very close to the bacterium, the number of new links formed between the bacterium and filaments, and the number of these bacterium–filament links broken. Above these are plotted the total number of bacterium–filament links. At the top, the net filament link force and the net filament collision force are given in picoNewtons (see Figure 1B), along with the total force. Some general trends aligned with pauses are apparent, such as decreased actin and link dynamics during a pause, but any characteristic biochemical or force response is obscured by the Brownian agitation of the bacterium.

**Figure 6:**
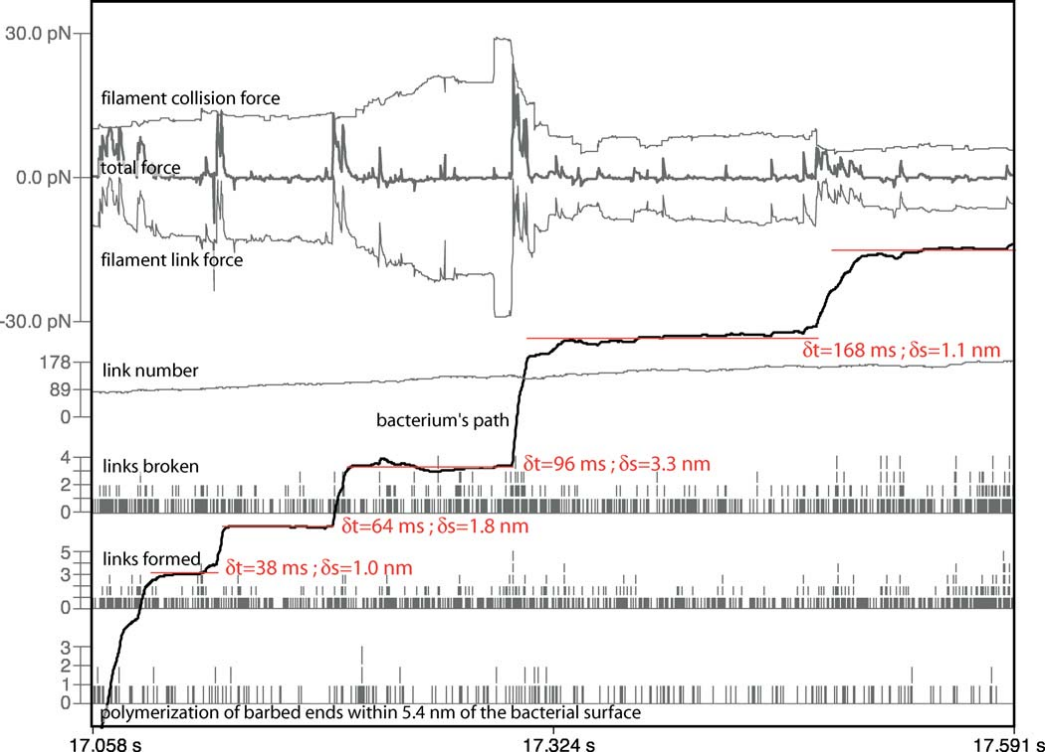
System Outcome Profiles for Several Adjacent Pauses—No Brownian Motion of the Bacterium Long pauses during this run without simulated Brownian motion of the bacterium (Brownian force multiplier of 0) reveal the force balance/imbalance conditions that cause pause–run behavior. System outcome profiles are shown for several adjacent pauses (red horizontal lines) with their duration (δt) and distance to the following pause (δs) displayed as reported by our line-fitted analysis. For a description of each of the entities plotted here, see the legend to Figure 5. Note that the bacterium pauses in its forward progress when the filament collision and link forces cancel each other; then, the collision forces tend to rise during a pause as filaments in the tail catch-up with the bacterium and generate new filament collisions. Runs are coincident with the breakage of many highly strained filament links which are quickly replaced by unstrained links; note that the filament link number does not change greatly during such an avalanche of link breakages and that it is steadily climbing over this time range in this particular simulation.

In Figure 5, we follow actin polymerization, link formation and breakage, link number, and path-directed forces for several sequential pause events during a single simulation. Owing to the stochasticity introduced by the Brownian simulation forces in this simulation, it is difficult to find trends in such single simulation profiles. We can learn more by turning off the Brownian simulation forces on the bacterium as has been done in figure 6. Now frequent long pauses are observed that clearly reveal the force relationships during pause initiation and termination. Both filament link force and collision force increase in magnitude synchronously during a pause, indicating resistance to forward progress by a population of ActA–actin filament links. The bacterium moves rapidly forward only when the total filament link force suddenly plummets. This sudden decrease in link force can only be attributed to a cascade of link breakage. This result indicates that the highly strained filament links that had balanced the filament collision force during a pause are rapidly exchanged for unstrained links when a pause ends.

**Figure 7:**
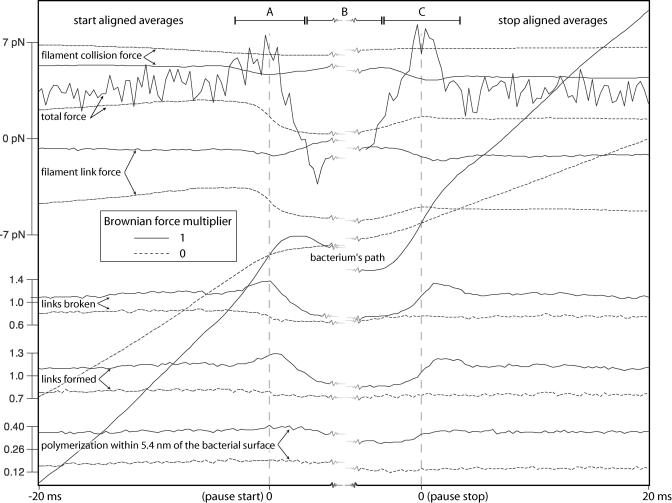
Anatomy of a Pause An ensemble of average system outcome analyses, with (solid lines) and without (dotted lines) simulated Brownian motion of the bacterium (see discussion on the relationship between applied Brownian simulation force and numerical time-step in the model description). These averages were compiled from pauses with duration greater than 10 ms, using 10,365 pauses with, and 4,358 pauses without, simulated Brownian motion (there are fewer, longer pauses without Brownian motion of the bacterium). Only sufficiently time-separated pauses contributed to these averages, so that the 10 ms preceding the pause start and the 10 ms following the pause stop are guaranteed not to include the effects of any adjacent pause. The Brownian simulation force trends can be read from the total force curve, which is only slightly offset by the link and collision forces in the case where Brownian movement of the bacterium is simulated (solid lines). Here, initiation of a pause follows a large forward-directed Brownian simulation force on the bacterium (segment A), which increases link turnover and produces a large population of synchronously strained links. Backward path-directed Brownian simulation forces (segment B) maintains the pause until, aided again by forward-directed Brownian simulation forces (segment C), the bacterium transitions back into a run. The Brownian simulation force trends can be read from the total force curve, which is only slightly offset by the link and collision forces. Without simulated Brownian motion of the bacterium (dotted lines), a pause is initiated and maintained when a population of ActA–actin filament links can resist the essentially constant total filament collision force. A pause terminates in this case when these links break en masse. Any individual pause in the averaged set of pauses might not demonstrate all of these response features.

The average of these system outcomes further clarifies pause causality and reveals differences between the two cases illustrated in Figures 5 and 6. From Figure 7 we see that pauses can occur with or without Brownian motion of the bacterium. But when we simulate the Brownian motion of the bacterium, we observe that pauses are correlated with an accidental sequence of similarly directed Brownian simulation forces (forward to initiate a pause, backward to sustain a pause, and again forward to break out of a pause). Note that any individual pause event in our average ensemble may experience only a subset of the correlated Brownian simulation force profile presented in Figure 7. On that figure we have partitioned this correlated Brownian simulation force into three temporal regions, labelled A, B, and C. An individual pause event might correlate with the Brownian simulation force sequence of A, or only A and C, or only B and C, or any other combination. Additionally, some pause events might be entirely uncorrelated with any Brownian simulation force trend. In other words, this averaging method reveals system trends that occur frequently, but need not be present in every contributing event.

We contrast the Brownian/no Brownian motion cases to better understand pause initiation, maintenance, and termination. Our most realistic simulation will incorporate effects from each extreme case. With simulated Brownian motion of the bacterium, we suggest the following causal temporal sequence for pause initiation, maintenance, and termination (with the caveat that most individual pause events will experience only a subset of this sequence):

A particularly large Brownian simulation force (or accidentally correlated sequence of forces) in the forward path direction causes an unusually rapid but small forward displacement of the bacterium (region A).

The steady-state rate of filament link turnover increases slightly as highly strained links break and are replaced by an ensemble of new links that all form nearly simultaneously in an unstrained state, thus creating a larger than steady-state population of coordinately unstrained links.

A particularly large Brownian simulation force, or correlated sequence of forces, opposite to the path direction forces the bacterium backward against the population of linked barbed end actin filaments; filament collision force increases, filament link force falls, and actin polymerization near the surface decreases. A pause ensues.

During the pause new filaments form and existing but distant barbed ends “catch up” with the bacterium, thus increasing the filament collision force forward which the links restrain. The pause terminates when a particularly large Brownian simulation force (or an accidentally correlated sequence of forces) in the forward path direction is sufficient to break a few of the strain-synchronized filament links. As these links break, the force stretching each remaining link increases, setting in motion an avalanche of cooperative link breakage and initiating a run.

We are justified in interpreting these correlations of Brownian simulation forces as causal because those forces are generated in our simulations so as to be random in direction and magnitude (representing a Gaussian distribution). Nothing in our model can cause such Brownian simulation force “accidents.” Their correlation with pause initiation or termination must therefore be causal.

Absent Brownian simulation forces on the bacterium, the system response throughout the course of a pause is very different. In this case, a pause occurs only when a population of synchronized filament links is able to balance the filament collision forces, which on average increase only slightly during a pause, until a cascade of breaking links allows the bacterium to run again. Judging from the shape of the step-size histograms in Figure 4, the generation of a set of strain-synchronized links that initiate a pause is likely a random event. That figure reveals a Poisson process-like distribution of step-sizes with weak or no Brownian simulation forces; moreover, the step termination (and therefore pause initiation) appears to occur with a constant probability through time. This should be contrasted with the case of simulated Brownian motion appropriate for an unconstrained bacterium, in which step-termination (pause initiation) is correlated with forward path-directed Brownian simulation forces.

The small amplitude of experimentally measured fluctuations of bacteria in vivo (Kuo and McGrath 2000) suggest that the simulations absent Brownian motion of the bacterium come closest to representing the biological reality; the coincidence of similarly directed Brownian movements is probably less important than the balance between filament–bacterium collision and link forces.

## Discussion

We have used our model to ask how L. monocytogenes motility is mediated by actin-mediated forces. Building a simulation from basic, well-understood structures and interactions, we have reconstituted bacterial motility in silico. Appropriate speeds and persistence of motion emerge, reproducing experimentally observed values. Additionally, our simulation yields as an emergent behavior the nanometer-scale saltatory motion reported by experimentalists. We can analyze details of the simulated bacterial trajectories to investigate characteristics of this saltation: what is the mechanism behind the stepping, and is there a favored step-size?

Our computational experiments lead us to conclude that the tethered-ratchet model is an inherent “pauser” with several important attributes. First, there is no characteristic step-size or pause length; shorter steps and pauses are more frequent than longer ones. Second, the intensity of Brownian agitation of the bacterium influences average pause duration and frequency, but this agitation is not necessary for persistent saltatory motion. Third, pauses start when a population of filament links happen to form nearly simultaneously with low strain to balance filament collision forces. Pauses end when those links catastrophically break.

To produce nanometer-scale pauses and runs, no special function need be attributed to the bacterial bound ActA protein, beyond an elastic linkage to actin filaments and some mechanism that prevents barbed end filament capping. Specifically, the ActA protein does not need a motor-protein-like stepping ability, nor need it act as a clamped-filament elongation motor (Dickinson and Purich 2002). Given the large number of filaments near the surface of the bacterium and the wide variation in angle of those filaments, it is not clear that we would expect any step-size, even if ActA were motor-like with a discrete working stroke.

The speed of motion during a run is variable, but it is mostly confined to a narrow range of speeds that depends on the parameter set (see Figure 4C). Pauses are a significant feature in our simulated trajectories; bacteria spend large fractions of their time paused (from 33% to 65% in the simulations presented here).

Lastly, we have explored the variation of key biochemical events and mechanical interactions during a typical nanometer-scale saltation, looking at both individual pause events and averages of many such events, in an effort to uncover the causal factors. We conclude that the tethered-Brownian ratchet model is an inherent pauser; forward motion is temporarily halted whenever a population of synchronously strained filament links balances filament collision forces. Different mechanisms cause pause initiation/termination with and without simulated Brownian motion of the bacterium. With simulated Brownian motion of the bacterium, we find that pauses events are largely driven by coordinated Brownian simulation forces: a series of forces in the forward direction helps establish a set of coordinately-strained links, forces in the backward direction can help maintain a pause, and lastly forces in the forward direction help break links to terminate a pause. Without simulated Brownian motion of the bacterium, we find that a coordinately strained set of filament links balances the filament collision forces and that a pause will ensue until those links break en masse. Formation of such a set of filament links is an accidental, but frequent, occurrence, explaining the shape of the step-size and pause duration histograms (see Figure 4).

That we find no characteristic step-size in our simulated nanoscale stepping constrasts with experimental results (Kuo and McGrath 2000; McGrath et al. 2003). Restricted by experimental noise, those researchers cannot see steps smaller than about 2.5 nm, if they indeed exist. Without those steps, about 30% of our simulated steps would be between 4 nm and 6 nm. We are presently sharing trajectories with the Kuo laboratory to directly compare model and experiment.

**Figure 8:**
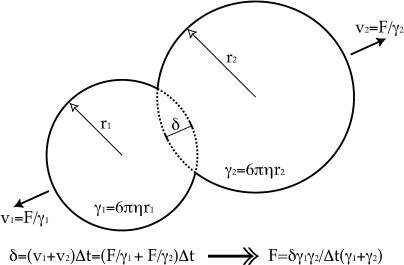
A Simple Collision Rule We calculate the magnitude, F, of equal and opposite forces applied to colliding objects such that they no longer collide after a time-step of δt. This force is calculated by considering the maximal distance, δ, of object intersection and the shape-based viscous drag, γ, for each object. In this example, we use Stokes' law for the viscous drag on spheres. We are assuming that the time-scale for a collision to resolve itself is much shorter than the discrete time-step used in the computation. We make similar calculations for more complex shapes and collisions.

Mogilner and Oster (2003) explore stepping behavior of their elastic tethered Brownian ratchet model and, for low filament tether numbers and particular capping rates and tether stiffness, observe step sizes similar to those reported by Kuo and McGrath (Kuo and McGrath 2000; McGrath et al. 2003). A step in that model occurs when one filament tether breaks and the remaining tethers all stretch in response to the new force balance. By constrast, steps in our model occur following a catastrophic breakage of many coordinately strained tethered filaments and are not highly dependent upon tethered filament number or capping rate. Because of the method by which we resolve collisions and strained links (see Figure 8), we do not prescribe the elastic properties of the filament–ActA links, and so our stepping is also independent of those values. Dickinson and Purich (2002) proposed a completely different mechanism for nanoscale stepping, involving a putative elongation motor that demonstrates approximately 5.4-nm stepping in simulations. In their model, most filaments are in compression while a few lagging filaments prevent the bacterium from moving forward. It is the “release and relocking” of a single lagging actin filament by the elongation motor that allows a 5.4-nm step. While the mechanisms are severely different, the basic behavior of this elongation motor model is similar to ours. Of the population of filaments interacting with the bacterium in our simulations prior to a step, a subset generates collision forces and a coordinately strained subset attached to ActA proteins balances those collision forces, resisting forward motion. The concurrent breakage of this linked subset allows a step forward, analogous to the “release and relocking” in the elongation motor model, though many more filaments are involved in our “release,” and the step distance before these filaments rebind to ActA, and thus “relock”, is not prescribed.

The computational analysis of L. monocytogenes motility described here represents a new tool that should be useful for understanding many complex subcellular systems. The construction of this computational model requires experimental measurements of the biological details in L. monocytogenes propulsion and actin dynamics in general. Only in the last several years have crucial biological details come to light, e.g., the role of Arp2/3 in filament branching, or the binding sites and functionality of the ActA protein. Additionally, the implementation of the model in silico requires significant computational power, now affordable in the form of clusters of “off-the-shelf” machines (we estimate use of 30 cpu years on a 2.8 Ghz Pentium 4 in the development and exploration of this model, 3.5 cpu years of which directly contribute to this report). Powerful object-oriented languages are also recently mature (we use Java™), making it possible to write computer code to implement such models. We believe that the confluence of detailed biological information and computational power/software heralds a new approach for understanding subcellular systems in which many thousands of simple biochemical and mechanical interactions lead to complex emergent behavior.

The biological systems in which this approach will be useful are, by definition, rich in detail. This complexity favors collaborations between modelers and the experimentalists who discover and quantify the molecular details without which this study would be specious. Creating a simulation environment that makes intuitive sense to experimentalists, i.e., one in which there is clear correspondence between biological entities and their modeled counterparts, greatly facilitates communication between modelers and biologists, and it ensures appropriate refinement of the model as new biological facts are uncovered.

There are many future refinements and research directions for this model. We can incorporate a more sophisticated representation of the actin hydrolysis cycle (Bindschadler et al. 2004) and include specifics of the interactions between ActA and proteins such as Ena/VASP. Recent work with ActA-coated beads (Cameron et al. 2004) characterizes the relationships between several biophysical parameters and motion initiation, speed, and persistence; these experimental findings can also be explored in silico. Extended with additional cellular components (e.g., a dynamic cortex, microtubules, motor proteins), the model might also be used to explore any of a number of cellular phenomena, including whole cell motility and cytokinesis. Our model, encoded in an object-oriented manner, is structured in a way that is strongly delimited by nature—so while we must still embrace approximation, we can minimize abstraction.

## Materials and Methods

A large set of differential equations determine how our state variables change with time. We solve these equations numerically, but not in a standard way because discontinuities in time occur frequently as objects collide suddenly and as objects suddenly spring into existence or disappear (due to new filament nucleation and depolymerization). To solve these thousands of differential equations, we divide time into discrete steps (typically tens of microseconds) balancing the necessities of capturing the system dynamics and accomplishing the simulation in reasonable human time (typically 3–5 d). At the beginning of each time-step, the biochemical events and forces experienced during the last time-step will have changed the state of the system. New collisions and link forces may have arisen, as well as new objects. Existing links may break if they experience excessive strain for several consecutive time-steps. Each explicit player thus experiences a net force vector; in the next step, we move each explicit player in this vector direction so as to reduce or eliminate the strain energy associated with its collisions and links. To accomplish this practically, we calculate the forces required to resolve each individual collision (or strained linkage) in a single time-step. Figure 8 demonstrates this calculation for a collision between two spherical bodies; a similar approach is taken for all pair-wise collisions or links. In brief, we sum all forces, attenuating all their magnitudes by the same factor without changing their directions, so that acting during the time-step they produce just enough displacement to separate objects that collided in the prior time-step. This process avoids prescription of elastic constants and is equivalent to proceeding through a series of quasi-static equilibria, a formally valid approach if the biochemical dynamics are slow relative to the resolution of force imbalance.

**Figure 9:**
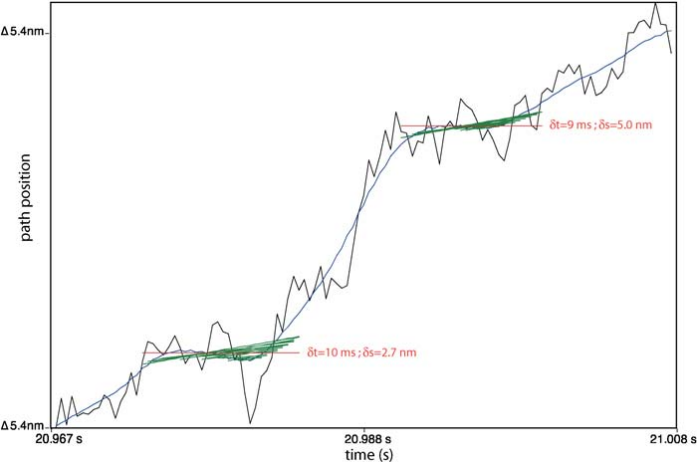
Slope-Based Analysis of Bacterial Trajectories A brief sample of a bacterial trajectory from one simulation run (black), a smoothed approximation to that trajectory (blue), line segments of near zero velocity fit to the smoothed trajectory (green), and the summary of a pause event assembled from those lines (red). Our analysis software seeks nearly horizontal segments of the trajectory (i.e., pauses), of maximal possible duration, in which excursions away from pause location lie with a jitter tolerance that we specify. Trajectories are curves in 3D space with curvature and torsion. To simplify the analysis, we use a path position variable (on the vertical axis), projecting each displacement onto the path tangent vector, instantaneously defined by the bacterial orientation. Then, we identify pauses in the resulting time series to specify displacement along the smoothed trajectory. Labels on each pause report pause durations, δt, and the displacement to the next pause (step-size), δs. Random thermal agitation forces act to buffet the bacterium, and every individual part, in our simulations; this is what makes the trajectories jagged.

Each individual computer run simulates bacterial motion for a period of up to many minutes. We run hundreds of such simulations and then statistically analyze the ensemble of runs. Fitting straight line segments to each trajectory and filtering those segments by slope (speed of motion) reveals that each simulated bacterial trajectory is composed of a sequence of pauses (of varying duration) separated by near-constant speed runs between pause locations. After we identify all the pauses, we measure the distances between adjacent pauses; these are the putative step-sizes. Histograms of pause duration and step-size, distilled from multiple simulations, then allow comparison with experimental observations and reveal whether there exists a preferred step-size or pause duration. Figure 9 shows a segment of trajectory data and the progressive stages of our line-fitting analysis.

**Figure 10:**
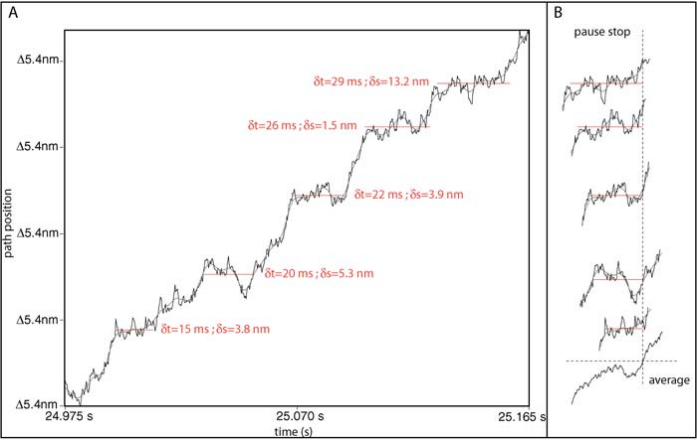
Calculating an Average System Response (A) shows a segment of simulated bacterial trajectory with five identified pauses shown in red; δt is the pause duration, and δs is the distance along the path to the next pause. To obtain the average response of any system outcome, we align the pauses at their stopping (or starting) points as shown in (B).

We average many thousands of pause events into portraits characterizing system behavior preceding, during, and following the typical pause. To do this, we align pauses, time and space shifting short sections of the path projected trajectories that span a single pause event so as to superimpose their starting or stopping points (Figure 10). These pauses are of different duration, so our average response will be most meaningful near the alignment point. To improve this analysis, we can also select and average only pauses of similar duration or create ensemble portraits from start-aligned and stop-aligned analyses.

Any trend remaining after the averaging of many thousands of events will reveal significant system behavior near the alignment point. Any individual event, however, might not exhibit all the trends revealed in such an average, so that the interpretation of these average profiles should be tempered accordingly.

Not all of the capabilities of our model have been enabled in the simulations contributing to this study. Our calculations show that the local depletion of the implicit players, due to their incorporation into a larger assembly, is not significant for the concentrations, rate constants, and geometries of this system (data not shown). Thus, we do not simulate the diffusion of any of the implicit players (proteins), but rather assume that each exists at a constant concentration (see Table 1). With this assumption, we need not accurately represent the depolymerization of the bacterium's comet tail in modeling the movement of the bacterium. (This depolymerization could otherwise have had an important role in regenerating depleted stocks of some implicit players.) We therefore depolymerize F-actin in the most computationally efficient way: we assume an artificially high pointed end depolymerization and ignore cleavage by ADF/Cofilin.

In addition, F-actin interacts with cellular components in vivo that are not explicitly represented in either the dendritic nucleation model or our simulations (e.g., with other cytoskelet al.filaments). Some of these interactions have the effect of locking down actin tail in cellular space. We approximate their effect with a time- and actin length-dependent application of adhesions that eventually fix F-actin and the actin tail in our simulation space.

## Supporting Information

Dataset S1 Psuedo Code for an Actin Filament (29 KB DOC).

Dataset S2 Steady-State Number of ActA–Arp2/3 Complexes on the Bacterium (52 KB DOC).

Video S1 One Actin Filament Interacting with the Bacterium A close-up look at the interaction of a single polymerizing filament with the bacterium. This filament has an artificially durable link with an ActA protein on the bacterium's surface; these links are typically very transient. The tip-clearance (drawn with a cyan line), the polymerization probability, the capping probability, and the Arp2/3 binding probability (set to zero for this demonstration) are reported at each simulation time-step.
(1 MB MOV).

Video S2 An Animated Simulation: Motion Initiation and Persistence An animation rendered from the output of one simulation of L. monocytogenes motility. Microscale hops, as opposed to the nanoscale steps we investigate in this paper, are apparent at this scale view. The bacterium induces an actin tail of variable density and demonstrates persistent motion.
(9.8 MB MOV).
