# Supplementary material for: In Silico Reconstitution of Listeria Propulsion Exhibits Nano-Saltation
Source: PLoS Biol. 2004 Nov 30;2(12):e412. doi: 10.1371/journal.pbio.0020412 (PMC532387; doi:10.1371/journal.pbio.0020412)
Supplement: Dataset S1 — (29 KB DOC). [file pbio.0020412.sd001.doc]

Appendix A: Psuedo-code for an actin filament

The following psuedo-code is a simplified representation of the object-oriented code defining the behavior of an actin filament. The characters “/*” define the beginning of a comment. Two coordinate systems are used: a fixed coordinate system denoted X-Y-Z, and a coordinate system attached to each filament denoted x-y-z (with x along the length of the filament). The methods shown to sum forces, move the filament, and simulate biochemical events could be performed in the order listed.

An_Actin_Filament {

/* variable declarations

position; /* a vector in 3-space, X-Y-Z position of centroid

orientation; /* a vector in 3-space, Euler angles defining orientation

force_sum /* forces on this filament in the fixed coordinate system

torque_sum /* torques on this filament in the fixed coordinate system

ATP_monomers; /* the number of monomer bound with ATP

ADP-Pi_monomers; /* the number with inorganic phosphate still bound

ADP_monomers; /* the number of hydrolyzed actin monomers

barbed_end_capped; /* a boolean flag , is a capping protein bound to the barbed end?;

pointed_end_capped; /* a boolean flag, is the pointed end capped?

Arp2/3_locations; /* list of Arp2/3 proteins bound to this filament

Sum_Forces {

For each bond with an ActA protein {

Add link force to force_sum;

Add torque from link force to torque_sum;

}

For each collision with another object {

Add collision force to force_sum;

Add torque from collision to torque_sum;

}

Move_Filament {

Translate force_sum and torque_sum from X-Y-Z to x-y-z;

Apply Newton’s laws in x-y-z {

ΣF = (force_sum in x-y-z) -μv = 0;

ΣT = (torque_sum in x-y-z )-μω = 0;

Solve for the translational(v) and rotational(ω) velocities;

}

Translate v and ω into X-Y-Z;

Increment the filament position and orientation, e.g. Δx = vΔt;

}

Filament_Growth {

If there is space near the barbed end {

Calculate probability, P+, of monomer addition ;

Get a random number, RND;

If (RND <P+) then {

Grow barbed end by one ATP actin monomer;

Remove one ATP actin monomer from local concentration;

}

}

Check pointed end growth and monomer dissociation from both barbed and

pointed end in a similar manner.

}

Filament_Capping {

If the filament not protected from capping by ActA {

calculate probability, Pcap, of capping the barbed end;

get a random number, RND;

if (RND < Pcap) then {

set barbed_end_capped to true;

remove on cap protein from local concentration;

}

}

}

}
