# Supplementary material for: In Silico Reconstitution of Listeria Propulsion Exhibits Nano-Saltation
Source: PLoS Biol. 2004 Nov 30;2(12):e412. doi: 10.1371/journal.pbio.0020412 (PMC532387; doi:10.1371/journal.pbio.0020412)
Supplement: Dataset S2 — (52 KB DOC). [file pbio.0020412.sd002.doc]

Appendix B: Steady-state number of ActA-Arp2/3 complexes on the bacterium.

Our approach is to find the diffusive flux of Arp2/3 proteins onto the bacterial surface and balance the kinetic on and off rates. There is such a high density of ActA on the bacterial surface that we assume each incident Arp2/3 protein forms an ActA-Arp2/3 complex. These complexes dissociate with the experimentally measured off rate of 0.6/s (Marchand et al. 2001) with an ActA we balance kinetic on and off rates to find steady-state. The transport rate of a protein to the surface of a sphere can be found from the Heat Equation as

where is the diffusivity of the protein, is the sphere’s radius, and and are the protein concentrations far from the sphere and at the sphere’s surface, respectively. Applying Dirichlet boundary conditions, we let be constant and set =0. Arp2/3 has a mass of about 220 kD, from which we estimate its volume, globular radius, and diffusivity. We use the rule of thumb that each kD of protein occupies a volume of 1.2 nm^3^ (Howard 2001).

Using the Stokes equation for the drag coefficient of a sphere and the Einstein equation we can estimate the diffusivity of Arp2/3.

and

where is the viscous drag, is the fluid viscosity (we use 3.0 mPa-s, Dembo 1989), is Boltzmann’s constant, is the temperature in Kelvin, and is the diffusivity. This diffusivity doesn’t account for the crowded, inhomogenous environment of the cell. In consideration of the work of Luby-Phelps (1987, 2000) we modify this “nominal” diffusivity by heuristically fitting a curve to experimental data (Figure 3, Luby-Phelps 2000):

with in nanometers. For Arp2/3, we find and thus . Using this value in our transport equation, we find an Arp2/3 flux onto a sphere of radius 0.35µm of

Which means that about 13,000 Arp2/3 proteins hit the surface of the bacterium for every micromole in solution. If we balance this “on rate” with the measured kinetic off rate, using the Arp2/3 concentration of Table 1, then we find the steady-state number of ActA-Arp2/3 complexes:

or . These complexes are ready to “grab” actin monomers and nucleate new actin filaments *de novo*.

With a similar calculation for G-actin we find hundreds of thousands of ActA-actin complexes at steady-state. We thus assume that ActA proteins are always associating with G-actin.
